# Barriers and facilitators of evidence-based management of patients with bacterial infections among general dental practitioners: a theory-informed interview study

**DOI:** 10.1186/s13012-016-0372-z

**Published:** 2016-01-29

**Authors:** Rumana Newlands, Eilidh M. Duncan, Maria Prior, Paula Elouafkaoui, Andrew Elders, Linda Young, Jan E. Clarkson, Craig R. Ramsay, for the Translation Research in a Dental Setting (TRiaDS) Research Methodology Group

**Affiliations:** 1 Health Services Research Unit, University of Aberdeen, Health Sciences Building, Foresterhill, Aberdeen, AB25 2ZD UK; 2 Dental Health Services Research Unit, University of Dundee, Park Place, Dundee, UK; 3 NHS Education for Scotland, Dundee Dental Education Centre, Frankland Building, Dundee, UK; 4 NMAHP Research Unit, Glasgow Caledonian University, Cowcaddens Road, Glasgow, UK

**Keywords:** Infection, Bacterial, Dental, Prescribing, Antibiotics, Drug resistance, Theoretical Domains Framework, Intervention design

## Abstract

**Background:**

General dental practitioners (GDPs) regularly prescribe antibiotics to manage dental infections although most infections can be treated successfully by local measures. Published guidance to support GDPs to make appropriate prescribing decisions exists but there continues to be wide variation in dental antibiotic prescribing. An interview study was conducted as part of the Reducing Antibiotic Prescribing in Dentistry (RAPiD) trial to understand the barriers and facilitators of using local measures instead of prescribing antibiotics to manage bacterial infections.

**Methods:**

Thirty semi-structured one-to-one telephone interviews were conducted using the Theoretical Domains Framework (TDF). Responses were coded into domains of the TDF and sub-themes. Priority domains (high frequency: ≥50 % interviewees discussed) relevant to behaviour change were identified as targets for future intervention efforts and mapped onto ‘intervention functions’ of the Behaviour Change Wheel system.

**Results:**

Five domains (behavioural regulation, social influences, reinforcement, environmental context and resources, and beliefs about consequences) with seven sub-themes were identified as targets for future intervention. All participants had knowledge about the evidence-based management of bacterial infections, but they reported difficulties in following this due to patient factors and time management. Lack of time was found to significantly influence their decision processes with regard to performing local measures. Beliefs about their capabilities to overcome patient influence, beliefs that performing local measures would impact on subsequent appointment times as well as there being no incentives for performing local measures were also featured. Though no knowledge or basic skills issues were identified, the participants suggested some continuous professional development programmes (e.g. time management, an overview of published guidance) to address some of the barriers. The domain results suggest a number of intervention functions through which future interventions could change GDPs’ antibiotic prescribing for bacterial infections: imparting skills through training, providing an example for GDPs to imitate (i.e. modelling) or creating the expectation of a reward (i.e. incentivisation).

**Conclusions:**

This is the first theoretically informed study to identify barriers and facilitators of evidence-based management of patients with bacterial infections among GDPs. A pragmatic approach is needed to address the modifiable barriers in future interventions intended to change dentists’ inappropriate prescribing behaviour.

**Electronic supplementary material:**

The online version of this article (doi:10.1186/s13012-016-0372-z) contains supplementary material, which is available to authorized users.

## Background

In 1945, Sir Alexander Fleming warned of the danger of over-reliance on antibiotics and the threat of bacterial resistance [1]. Seventy years later, inappropriate antibiotic use contributes to an increasing risk of antimicrobial resistance [2, 3] and the rising levels of resistance have become a recognised threat to global health and a challenge to most healthcare systems [4, 5]. General dental practitioners (GDPs) prescribe antibiotics regularly for the management of dental infections, when most infections can be treated successfully by removal of the source (i.e. extraction of the infected tooth) [6]. Dental prescribing accounted for around 9 % of total antibiotic prescribing in Scottish and English primary care in 2013–14 [7, 8]. The dentists’ chosen treatment is often guided by personal experience and knowledge, and in many cases, antibiotics are prescribed inappropriately to patients who present with dental emergencies [9–11].

Published guidance to support GDPs to make appropriate prescribing decisions recommends that bacterial infections should be treated with local measures (e.g. for dental abscesses, drain any pus present by extraction of the tooth or through root canals and attempt to drain any soft-tissue pus by incision) in the first instance and that antibiotics are required only in cases of spreading infection (e.g. cellulitis, swelling) or systemic involvement (i.e. fever, malaise) [12, 13]. Despite the introduction of the guidance, there continues to be wide variation in dental antibiotic prescribing [7, 14].

There is ample evidence that the implementation of guidance by health care professionals (including dentists) is variable, and understanding how to improve implementation is limited [15–18]. In order to develop successful strategies to reduce the variation in implementation of guidance, an understanding of the types of barriers faced in healthcare is required along with a tailored approach to overcome these barriers and encourage behaviour change [19].

Previous studies have explored the antibiotic prescribing knowledge of dental professionals [20] and their beliefs and attitudes towards antimicrobial use and resistance [21] but, to date, no research has been undertaken to identify the barriers and facilitators of evidence-based management of patients with bacterial infections among GDPs in the United Kingdom (UK). In order to facilitate understanding of the barriers and facilitators of evidence-based practice among GDPs, a theory-informed interview study was conducted using the Theoretical Domains Framework (TDF) [22]. This was performed as part of the Reducing Antibiotic Prescribing in Dentistry (RAPiD) study [23], a 12-month partial factorial cluster randomised controlled trial (RCT) conducted in National Health Services (NHS) General Dental Practices across Scotland comparing the effectiveness of individualised audit and feedback (A&F) strategies for the translation into practice of the Scottish Dental Clinical Effectiveness Programme (SDCEP [12]) guidance on antibiotic prescribing [23]. The aim of this interview study was to identify barriers and facilitators of managing patients with bacterial infections using local measures rather than prescribing antibiotics and to identify future intervention options.

## Methods

### Study design

Semi-structured one-to-one telephone interviews were conducted. Section 1 of the interview related to participants’ experiences and responses to the RAPiD trial audit and feedback intervention and were based on the Consolidated Framework for Implementation Research (CFIR [24]). Section 2 of the interview used a topic guide based on the TDF to explore the factors influencing GDPs’ management of patients with bacterial infections.

### Materials

The TDF has 14 domains [25]. It allows investigation of the potential influences on health professional behaviour inherent to implementation of evidence-based practice [26, 27] and also informs the development of behaviour change interventions [28–31]. The TDF-based interview topic guide [23] was developed and refined through pilot testing with two dentists (known to the study team) and discussions within the research team. Open-ended and closed-ended questions were used and the number of questions ranged from one to three for each TDF domain. Prompts relating to the domain ‘Goals’ were not included due to the overlap with prompts from Section 1 of the interview (the CFIR-based prompts). Follow-up prompts were included when necessary to address specific constructs within the domains.

### Participants and recruitment procedure

GDPs working in the 795 General Dental Practices randomised at the beginning of the RAPiD trial, and who continued to work in a trial practice at the time of the 6-month intervention, were eligible to take part in the interview. Recruitment commenced in December 2013 after participants from the RAPiD trial had received the 6-month intervention. Potential participants were categorised into three groups (low, medium and high) according to their level of antibiotic prescribing during the year prior to the RAPiD trial interventions. A sampling frame of 300 GDPs was randomly selected from the study population, ensuring representativeness based on Health Board, practice prescribing level and practice size. From this sampling frame, 100 GDPs were randomly selected from the study population to receive notification by a postal letter that they might be contacted to take part in an interview. GDPs were given the option to opt-out and to not be contacted further. In addition, this notification was sent to nine GDPs who had contacted the TRiaDS (Translation Research in a Dental Setting) office after receiving intervention materials and indicating that they would be interested in being interviewed at a later date. Within 2 weeks of their notification, GDPs were purposively sampled and contacted, by telephone, by a member of the research team to discuss any queries and to ascertain their willingness to take part in the study. Diversity variables tracked throughout recruitment in order to maximise sample representativeness were as follows: trial group allocation; Health Board; practice prescribing level (low/medium/high); practice size; practice location (urban or rural); and deprivation. Further detail on the sampling and recruitment strategy is included in the study protocol [23]. A mutually convenient time for a telephone interview was arranged with those GDPs expressing an interest in taking part. No further contact was made with GDPs who did not wish to be interviewed. GDPs, who took part in the interview, were eligible to claim an honorarium of £40. The interview process was ceased after the 30th interview when no new themes emerged [32].

### Data collection

All interviews were conducted by an academic researcher [RN], and the participants were made aware of the interviewer’s non-dental background. Informed verbal (recorded) consent was obtained from all participants prior to interview, and confidentiality of the participants was assured. The mean interview length was 23.5 min (range 10.2–37.5 min). The interviewer knew in advance the participant’s dental practice trial group allocation but was not aware of the individual dentist’s prescribing behaviour or the prescribing profile of the dental practice. Interviews were transcribed verbatim by a professional transcription service. The quality of each transcript was checked against the original recordings [RN], and fidelity of the use of the topic guide was checked by another researcher [MP] who listened to the first two interview recordings. In accordance with the principles of good clinical practice, all data were stored in a password-protected computer and any identifiable data was removed from the transcripts. Access to data was restricted to appropriate members of the research team.

### Analysis

A coding guide was developed based on the published definitions and constructs of the TDF domains [25] and agreed by the research team [RN, ED and MP] for the purpose of consistent coding (Additional file 1). Transcripts (i.e. entire interviews) were imported into the qualitative data analysis software package NVivo 10 (QSR International) [33], and excerpts were coded into the main domains of the TDF [RN] using theory-based content analysis [34]. In the next stage, utterances (or ‘specific beliefs’ [26], including both barriers and facilitators) were collated, defined and grouped into sub-themes under each domain. A second researcher coded 10 % of the transcripts independently to ensure fidelity of the coding guide [MP]. All coding was discussed and agreed by three researchers [RN, ED and MP; two of whom have expertise in the TDF]. Further analyses were also performed to compare interviewees by demographic variables [i.e. prescribing level, multi-/single-handed practices, Scottish Index of Multiple Deprivation (SIMD) and years of experience].

Analysis then progressed through two stages, outlined below.

#### Identifying what needs to change when dentists manage patients with bacterial infections

In order to establish the barriers and facilitators of the target behaviour (i.e. managing patients with bacterial infections using local measures rather than antibiotics), criteria were developed to determine which domains of the TDF were ‘relevant’ for this behaviour (as per previous TDF research) [30, 35], i.e. which domains contain targets for change. Relevant domains were those where there was either (i) variation in reported behaviour/beliefs or (ii) reported barriers/facilitators to carrying out local measures rather than prescribing antibiotics.

#### Intervention development phase

The list of relevant domains, identified through stage 1, was examined in order to identify which might be the priority domains to target for future intervention efforts. Domains were prioritised based on the frequency with which they were coded (and thereby those domains were most often identified by the participants as containing targets for change). Priority domains were defined as those where 50 % or more interviewees provided content that was coded. Priority domains were then mapped onto components of the Behaviour Change Wheel (BCW) [36] framework (a synthesis of 19 frameworks of behaviour change) using ‘intervention functions’ [36]. Intervention functions are the mechanisms by which an intervention can change behaviour [28, 29, 36], for example, ‘education’ is an intervention function that works through increasing knowledge or understanding, ‘persuasion’ is the use of communication to induce positive or negative feelings or stimulate action and ‘incentivisation’ works through creating the expectation of reward.

### Ethics

The East of Scotland Research Ethics Service considered the RAPiD trial protocol and confirmed that it did not require ethical review or approval by an NHS REC (Ref: 11/GA/229). In addition, the protocol was submitted to the NHS Research Scotland Permissions Coordinating Centre and reviewed by the Tayside Medical Science Centre (TASC) Research and Development (R&D) office. They classified the RAPiD trial as service development/audit and confirmed that it did not require R&D registration, formal review, or approval.

The RAPiD trial’s International Standard Randomised Controlled Trial Number (ISRCTN) registration is ISRCTN 49204710.

## Results

### Characteristics of participants

Sixty-one of the 109 GDPs notified of the study were contacted and invited to take part. For various reasons (e.g. no longer working at the practice, maternity leave, orthodontist, too busy, multiple attempts to arrange a time), it was not possible to set up an interview with 31 of the GDPs contacted, thus giving a study response rate of 49 % (30/61). A comparison between the 30 GDPs recruited and the 31 who did not take part revealed no differences across most of the demographic variables (Health Board, practice size, trial arm and deprivation). However, there was a greater proportion of GDPs from rural areas in the interview group compared to those who did not take part, and their prescribing rate profiles also differed. Approximately 20 % of the GDPs interviewed were low prescribers, compared to almost 60 % of the GDPs who were not interviewed. Similarly, in the interview group, a smaller proportion of the practices (3 %) had all low prescribing GDPs, compared to over 25 % of the practices of those who did not take part.

Of the 30 participants, 23 were recruited from the intervention arms and 7 from the control. Twenty were male and ten were female. All but six participants qualified from dental schools in the UK, and the mean time since graduation was 20 years (range 3–40 years). Most participants (*n* = 26) reported treating both NHS and private patients, with four treating NHS patients only. There were 14 medium, 9 high and 7 low prescribers. No differences were found when interview content and themes were compared across demographic variables.

### Identifying what needs to change when dentists manage patients with bacterial infections

The theoretical domains that were judged to be relevant (i.e. where there was either (i) variation in reported behaviour/beliefs or (ii) reported barriers/facilitators to carrying out local measures rather than prescribing antibiotics) were ‘behavioural regulation’, ‘social influence’, ‘reinforcement’, ‘environmental context and resources’, ‘beliefs about consequences’, ‘beliefs about capabilities’, ‘memory attention and decision process’, ‘optimism’ and ‘emotion’. A total of 16 sub-themes from these relevant domains were identified and are discussed below (and displayed with illustrative quotes in Table 1).

**Table 1:** Summary table of relevant domains

Domain (descriptions) of the TDF [25]	Sub-themes	Frequency (utterances/participants)	Sample quote (with participant number)
Behavioural regulation	*CPD programmes are required.*	*56/29*	I think perhaps it might be good to have a course whereby we can probably get a better understanding of the actual, the guidelines (SDCEP), I think that would useful in the first place. (11360)
			I suppose a course on time management I might consider. I would heartily endorse TRiaDS setting up an online course for it, on both antibiotic prescribing and an online course on time management, especially as it relates to that, and having it that you get an education allowance for doing it, and that you sit and do it yourself at home. (10841)
	*Updated SDCEP or other guidelines are required.*	*35/23*	I tend to follow the SDCEP books to be honest when it comes to prescribing. I tend to find it’s the easiest thing to hand, it’s short, it’s succinct, it’s clear so I always go with that one. (10469)
			If they (SDCEP) publish a guideline, like a flowchart, this is what walks in the door; this is what the gold standard would be. That would be quite useful actually. (10968)
	Audit can reduce antibiotics prescribing practice.	8/7	I use prescriptions when I think they’re necessary. I’m not likely to change that drastically. (11355)
			Yes, I think it (audit and feedback) probably can change prescribing practice, yes. I have participated in one before, I think that certainly helps. (10545)
	Arranging appropriate emergency slots would be difficult.	11/9	I mean, you could put time aside each day to see emergency patients, but sometimes it’s difficult to fill in and it’s empty. Yes, we should have a time during the day, and say, “Right, that’s where emergency goes, that’s when we do these sorts of things”, but in practice, you know, what you set out to do in, doesn’t always happen. It’s always changing. (11006)
			I suppose if you … we’ve tried it, with a lack of success, is where you actually create some times of a day for emergencies and so that you’ve got that time available to do it, but we found it quite unsuccessful. (10841)
Social influence	*Patient behaviour or demands influence my prescribing behaviour.*	*81/25*	While on paper it sounds like a very good thing to refuse giving them an antibiotic if the patient actually is absolutely adamant they are not having actually any active treatment carried out it’s very difficult not to give them an antibiotic. (10968)
			I tend to find where I get frustrated with the prescribing when you feel that you can’t do the treatment that is best for the patient but, again, if you’ve not got consent then what can you do? (10469)
Reinforcement	*There are no incentives to conducting local measures.*	*39/24*	I would say minimal, but the time taken to do it will probably outweigh the actual fee that we get. (13602)
			In fact it’s (financial factors) one of the few times you don’t ever have to really think about it. If somebody’s got a really nasty infection I think it’s one of the few times you don’t think about it because if you can get it right it’s the one time people go out and say, “Thanks very much”. (10390)
Environmental context and resources	*Lack of time plays a big part in managing bacterial infections.*	*58/20*	It can depend with being in a busy general dental practice I think you are sometimes under time pressure and I’m going to be honest there have been times where you’ve been totally backed up and I think to be able to give the patients the best treatment instead of diving in and doing what could be deemed a difficult surgical extraction; I have prescribed so that I can rebook the patient for a time that I would manage the situation to the best of my abilities. (10469)
			Well, I would attempt to persuade them to have the treatment carried out, but some of these patients will not respond to that, and given the time constraints that we have treating patients, I often resort, I have myself resorted to just giving them the antibiotics that they’re looking for. (10545)
Beliefs about consequences	*Local measures involve a lot of time to conduct it successfully.*	*24/16*	… the fact that you’re going to run late and make other people wait so people get angry. (106000)
			Well, the downside is obviously the time factor. It takes a lot more time to carry out a local measure in comparison to writing a prescription for antibiotics. (11095)
	*Local measures occasionally make things worse.*	*36/16*	Occasionally local measures makes situation worse. I’ve certainly seen that. If you’re doing root canal work and you open it up and if it’s been dead for a long time and you open it all up the infection actually can get much more painful over the next few days. And, of course, then they (patients) blame it on us for doing the root canal work. (10968)
			I mean you are actually dealing with the infection which is there with local measures, as opposed to just prescribing antibiotics. I don’t think antibiotics ever work particularly well. (10935)
*Beliefs about capabilities*	It is difficult to apply local measures in phobic patients.	13/10	I tend to find that difficulties comes part and parcel with the anxiety as well. Patients who are more anxious I think dentists struggle to get those numbed up more regularly than others so I would say that sometimes is an issue. (10469)
			The non-attenders or infrequent attenders hate coming to the dentist and they tend to be more frightened, they tend to be more nervous. They’re the ones that are going to be resistant, they won’t allow you or they don’t want you to do local measures. So, you know, they tend to be a more difficult group to manage. (13474)
	It is sometimes difficult to numb the patient and conduct local measures successfully.	16/10	It’s difficult when there is, you know, inflammation, if the gums are swollen and when there is a pulpitis it is difficult to numb the patient. (10320)
			The lower jaw you can freeze up with a single injection; it’s called an alveolar block. It freezes it right at the very back so there doesn’t tend to be the same issues, so there’s no transmissions from the teeth, whereas with the upper teeth it is very much a localised injection round an infected area, which sometimes just doesn’t work. (11360)
	Time allocated per patient is not long enough for conducting local measures.	6/5	Time management is much more of a problem, yes. Because most of the time when we get people with infection problems, they really don’t have any time allocated to treat them. It’s not always possible to allocate the time required to treat them when, at the correct time. (11355)
			I see people sometimes, every five minutes, every ten minutes, you get less time than a GP does, to see somebody, to see what their problems is, to diagnose their problem and to try and treat them, you know? (13474)
*Memory attention and decision process*	Patients co-operation, consent influence my decision.	25/14	It’s kind of assessing the patient, how much they’re going to put up with. It’s alright saying okay, well we’ll drain this abscess but if you’ve got a very, if you’ve got a very anxious patient then sometimes, I, you know, I might try antibiotics just to calm the area down first. (10560)
			Just on the individual. If they can sit for the procedure and they’re happy to have something done that day then that’s absolutely fine. If I’ve got time then I’ll definitely do it and if they don’t want it then I’ll still try and persuade them that they should get this done. If they won’t sit, as in they can’t co-operate or they won’t co-operate, then it might be that I have to give them a prescription and get them back in when it’s all sorted out. (106000)
	Types of patient influence my decision.	11/9	I have to say, quite often I will prescribe an antibiotic as a fallback, we’re quite a rural area and quite a lot of patients come quite a long distance, so I’m not averse to handing out a prescription and telling them to get it filled and to have it in reserve, and if, I don’t know, about a day or two, they either phone up to get advice or just start taking the antibiotic. I know that’s probably not the guidelines, but in a more remote area, I think it saves people possibly a round trip of 100 miles to come and see us, when they’re already systemically unwell, is probably a good idea. (13308)
			Well one thing would be if it’s not my patient, if it’s somebody that I don’t know well, but my colleague does, who normally treats them. And this works both ways, and I’m sure that they’re more likely to prescribe an antibiotic in that case, when it’s somebody who usually sees one of our colleagues. (11156)
*Optimism*	Not sure if local measures will solve the issues successfully on their own.	5/5	I think I get worried that the patient won’t respond as I would like to after doing local measures. I’m not worried that I’m not doing the right thing, I’m just worried that the patient won’t answer to the things that I’ve done …(13388)
			I suppose that an antibiotic, you could argue, is like belt and braces, you know if it’s like a Friday or something then perhaps it’s not so good because, you know, if local measures don’t work over the weekend. (10868)
*Emotion*	I feel anxious about letting somebody go without antibiotics.	8/5	Yes, the anxieties about letting somebody with a facial swelling go out without antibiotics… I felt that holistically it was better to make sure that he was okay for going to Dubai. (10841)
			I don’t know but just around holiday times and that’ll be all the people that I get a bit worried about, they are heading off on their June holiday and I don’t want them to suffer, I want them to have something (antibiotics) in reserve, so I know, probably you’re not supposed to do that. (13308)

Note: domains/sub-themes in italics are categorised as priority (high frequency or ≥50 % interviewees discussed) for future intervention efforts

The participants identified ways to perform the target behaviour appropriately under behavioural regulation. Twenty-one participants mentioned that ‘Continuing Professional Development (CPD) programmes are required’ and suggested content such as time management, an overview of SDCEP prescribing guidance, discussion on what works in real life and some hands-on sessions on “tricky parts” (e.g. how to insert anaesthetics on upper tooth). They also expressed a preference for online CPD courses with stipends. Eight other participants mentioned that their experience and dentistry degree were enough to manage bacterial infections effectively. In relation to attitudes to guidelines, 16 participants mentioned that the SDCEP guidance is a handy, easy-to-follow and useful resource, whereas 7 others felt that ‘updated SDCEP or other guidelines are required’ such as providing a gold standard flowchart and/or a step-by-step guide on managing bacterial infections. Five participants believed that ‘audit can reduce antibiotic prescribing practice’. Two participants suggested that audit may not change their behaviour because they only write prescriptions when necessary and therefore their practice is “not going to change drastically”. Seven participants mentioned that arranging appropriate emergency slots as a strategy to address “chaos” (delay to routine appointments) produced by emergency patients ‘would be difficult’. Treating patients with local measures, rather than merely providing a prescription for antibiotics, requires a longer emergency appointment, and implementing dedicated emergency slots is not easy in practice because every day is different. In addition, because they are paid an individual fee for each item of treatment provided, if the emergency slot is not used, they forego the fees they would otherwise have earned. Some participants mentioned having tried unsuccessfully to implement dedicated emergency slots within their practice instead of relying on emergency patients’ “sitting and waiting”. However, one participant believed that implementing emergency slots would be helpful, and another participant had already implemented a 30-min emergency slot per day, which was mentioned as being enough time to treat emergencies in their own, as well as colleagues’ patients. ‘Patients’ behaviour or demands influence my prescribing behaviour’ under social influences was the most widely discussed potential influence on the target behaviour. Fifteen participants reported being influenced by patients while ten others were resistant to patients’ behaviour or demands. Demands for antibiotics were reported to come from patients about to go on holiday or those who are phobic/anxious. Not prescribing antibiotics was described as “tricky” when faced with pressure from patients and also when patients do not give consent for local measures.

Reinforcement was identified as a relevant domain because of the diversity of views expressed with regard to financial/non-financial incentives to conducting local measures. Sixteen participants mentioned that ‘there are no incentives to conducting local measures’ because the time taken to conduct appropriate local measures was believed to be incommensurate with the actual fee received per treatment. Therefore, appropriate remuneration was suggested for GDP practising under NHS. However, eight other participants mentioned incentives to conducting local measures including “it saves repeat visits”, “patients experience immediate relief”, and “appreciate your help”.

Another widely discussed concern was how a ‘*Lack of time plays a big part in managing bacterial infections*’ under environmental context and resources. Fourteen participants mentioned that due to lack of time, appropriate communication with patients to encourage them to accept local measures was challenging. Time management was mentioned as the “biggest bug bear in dentistry” and “highest up the scale of barriers” for managing patients with bacterial infections, and, due to lack of time, antibiotics were often prescribed as a “quick fix to help in that situation and help the dentist time-wise”. Six other participants who reported no issues with time management either treated a manageable number of patients every day or tried not to be affected by time pressures, and therefore each condition was treated appropriately rather than prescribing antibiotics only.

Various positive and negative consequences of conducting local measures were also reported in the beliefs about consequences domain. Sixteen participants mentioned that ‘local measures involve a lot of time to conduct it successfully’ compared to the time required to write prescriptions. The participants noted that if local measures were performed for each and every emergency patient, appointments would definitely overrun and this would upset subsequent patients, which was mentioned as not good for their business. Six participants, under the same domain, discussed negative consequences of local measures, such as ‘local measures occasionally make things worse’. They noted that because pain can increase as a result of local measures, patients may blame the dentist for the pain and subsequently may lose confidence in the dentist’s ability and may not come back to the practice. In contrast, ten other participants under the same sub-theme mentioned that local measures solve problems as opposed to giving antibiotics which may only result in re-occurrence of the problem at a later date, for example, patients get immediate pain relief by drainage of the tooth and it also makes the next stage of the treatment easier.

Perceptions about control over own behaviour (such as whether successfully performing the target behaviour is within or out with their control) were coded within beliefs about capabilities domain where a number of barriers were identified. Ten participants mentioned that ‘it is difficult to apply local measures in phobic patients’, where phobic patients were described as usually poor/irregular attenders. Children were also mentioned as often being frightened of any local measures. Referrals to hospital and access to sedation were discussed as solutions to treat these types of patients appropriately. Five participants stated that the ‘time allocated per patient is not long enough for conducting local measures’ as they usually see patients in every 5 or 10 min, which was not considered long enough for both diagnosing a bacterial infection and conducting local measures. Ten participants stated that ‘it is sometimes difficult to numb the patient and conduct local measures successfully’. Other difficulties mentioned were diagnosis issues (i.e. determining which tooth is creating problem), presence of infection (i.e. cannot inject anaesthetics due to the chance of spreading infection), difficulty in accessing the problem tooth (i.e. due to large swelling and trismus), patients who are too uncomfortable to co-operate or who do not respond to anaesthetics and difficulties with anesthetising an upper tooth due to its position. Therefore, in some cases, either local measures were not attempted or the clinician had to stop in the middle of treatment and consequently antibiotics were prescribed to reduce pain and inflammation.

Factors influencing dentists’ decision processes for managing bacterial infections were expressed in the memory attention and decision process domain. ‘Patients’ co-operation and consent influence my decision’ was mentioned by 14 participants and was further discussed as related to time and patients’ demand or behaviour issues. If patients did not initially give valid consent for local measures and there was not enough time to persuade them to accept local measures or if the patient was in too much pain to co-operate, dentists felt “morally obliged” to treat the infections with antibiotics rather than “nothing”. Within the same domain, ‘Types of patient influence my decision.’ was discussed by nine participants, for example, if the patient was new or a poor/irregular attender (i.e. who only turns up in pain), antibiotics were not provided, and local measures were conducted straight away. But if it was a colleague’s patient, a definitive treatment was left to be conducted by patients’ own dentist, and antibiotics were prescribed. Moreover, if patients were travelling abroad or visiting from remote areas, antibiotics were often prescribed as a “fall back” to make sure their holiday was not spoiled by an abscess and to avoid their revisit, respectively.

Five participants expressed their pessimism stating how they were ‘not sure if local measures will solve the issues successfully on their own’ under optimism. Therefore, when they were uncertain about the effect of local measures (e.g. on a Friday, if the patient was going abroad or if the patient came from a remote area), antibiotics were prescribed as a “belt and braces” approach. Similarly, five participants stated that ‘I feel anxious about letting somebody go without antibiotics’ under emotion due to the presence of swelling or pain following local measures, if the patient had declined local measures or was going on holiday.

No barriers were reported in the domains of ‘knowledge’, ‘intentions’, ‘social professional role and identity’, ‘skills’ and ‘goals’. All participants had knowledge of the SDCEP prescribing guidance and the recommendation to manage bacterial infections using local measures as a first instance. They expressed their conscious decision to perform the target behaviour in the intentions domain whereas in the social professional role and identity domain, the participants discussed that local measures are a part of their perceived routine jobs. The participants also believed that they had the necessary skills required for conducting local measures, such as appropriate communication with patients and basic dentistry skills. However, they expressed interest in attending CPD courses to address difficulties associated with local measures. No specific questions related to goals were asked in the interview.

### Intervention development phase

The domains categorised as high frequency (≥50 % interviewees) and therefore as priorities for future intervention efforts were ‘behavioural regulation’, ‘social influences’, ‘reinforcement’, ‘environmental context and resources’ and ‘beliefs about consequences’ (Table 1). The priority domains were mapped onto intervention functions (i.e. categories of the ways in which an intervention can change behaviour) from the BCW using published matrices [36] as outlined in Table 2. Eight intervention functions were identified as methods by which future intervention efforts could change GDP antibiotic prescribing behaviour; ‘training’, ‘enablement’, ‘modelling’, ‘education’, ‘restriction’, ‘environmental restructuring’, ‘incentivisation’, and ‘coercion’.

**Table 2:** Priority domains mapped to intervention functions

Domains identified as priorities (i.e. high frequency and existing barriers to be targeted)	Targets for change	Intervention functions [36]	Intervention function definition [36]
Behavioural regulation	CPD programmes are required.	Training	Imparting skills
	Updated SDCEP or other guidelines are required.	Enablement	Increasing means/reducing barriers to increase capability (beyond education and training) or opportunity (beyond environmental structuring)
		Modelling	Providing an example for people to aspire to or imitate
		Education	Increasing knowledge or understanding
Social influences	Patient behaviour or demands influence my behaviour.	Restriction	Using rules to reduce the opportunity to engage in the target behaviour (or to increase the target behaviour by reducing the opportunity to engage in competing behaviours)
		Environmental restructuring	Changing the physical or social context
		Enablement	(See above)
		Modelling	(See above)
Reinforcement	There are no incentives to conducting local measures.	Training	(See above)
		Environmental restructuring	(See above)
		Incentivisation	Creating the expectation of reward
		Coercion	Creating the expectation or punishment or cost
Environmental context and resources	Lack of time plays a big part in managing bacterial infections.	Training	(See above)
		Restriction	(See above)
		Environmental restructuring	(See above)
		Enablement	(See above)
Beliefs about consequences	Local measures involve a lot of time to conduct it successfully.	Modelling	(See above)
	Local measures occasionally make things worse.	Education	(See above)
		Persuasion	Using communication to induce positive or negative feelings or simulate action

## Discussion

This is the first study to identify barriers and facilitators of evidence-based management of patients with bacterial infections among GDPs. This study applied a systematic process to identify potential intervention options to improve GDPs’ evidence-based practice. The TDF-based interviews identified a range of influences on the target behaviour (i.e. managing patients with bacterial infections using local measures rather than antibiotics). Five domains were identified as priority targets for future intervention efforts: ‘social influences’, ‘environmental context and resources’, ‘beliefs about consequences’, ‘reinforcement’ and ‘behavioural regulation’.

The findings suggest that all participants have the knowledge required for evidence-based management of bacterial infections but reported difficulties following this on a day-to-day basis due to patient factors and time management. Lack of time was found to impact the management of bacterial infections and influenced dentists’ decision processes with regard to conducting local measures, their beliefs about their capabilities of overcoming patient influences and their beliefs about over-running appointments as well as there being no incentives to conduct local measures. However, it was identified that the time issue was mainly due to inadequate time allocated per patient and the lack of emergency slots allocated in the booking system. Therefore, a time management course was not only suggested for dentists but also for practice managers and receptionists who book appointments and initially deal with patients.

Some participants mentioned using the SDCEP guidance mainly while prescribing antibiotics (e.g. to find information on first-line antibiotics, dosage) whereas some struggled to remember the content. Therefore, an overview of the SDCEP guidance was suggested as a potential CPD programme. Although dentists felt they had the basic skills required to conduct local measures, they suggested some CPD training to address “tricky parts” associated with local measures, for example, how to administer anaesthetic in an upper tooth and how to lance an abscess when swelling is present.

No differences in interview content and themes were found across interviewees with different prescribing rates. One potential reason behind this null finding could be that the difference in prescribing rates observed reflects some differences in practice population characteristics and that GDPs are prescribing appropriately for their own particular practice population.

A similar study investigating Welsh GDPs’ perceptions of antimicrobial use and resistance also reported that time pressure had a big impact on their prescribing decisions for patients with acute dental conditions. This led to the suggestion that adding incentives for conducting operative treatment under the NHS contract may improve GDPs’ behaviour. A further suggestion included raising public awareness of antibiotic resistance to reduce patients’ expectations of antibiotics [21]. However, research [37] suggests that there is a discrepancy between the actual number of patients requesting antibiotics and clinicians’ perceptions about the number of patients expecting antibiotics; therefore, the scale of this problem would need to be assessed during any intervention development.

Previous interventions that have successfully improved prescribing behaviour among GDPs include clinical audit [38, 39] and providing an overview of a guideline for managing acute dental pain [40]. Whether these interventions resulted in long-term changes in GDPs’ behaviour is unknown. The DREAM trial, another recent study, developed a complex educational intervention to improve decision-making processes with regard to antibiotic prescribing among German GDPs [41]. The feasibility and effectiveness of this intervention are still to be evaluated. The domains identified as priority in our study suggest a number of intervention functions through which future interventions could be successful in changing GDPs’ antibiotic prescribing for bacterial infections. For example, imparting skills through training, providing an example for GDPs to imitate (i.e. modelling) or creating the expectation of a reward (i.e. incentivisation) could prove effective methods for intervention efforts. However, further consideration and careful piloting would be needed to ensure that any intervention functions utilised were affordable, acceptable and practical.

### Strengths and limitations

The management of patients with bacterial infections is regarded as a sensitive topic, and therefore, some participants’ responses may be biased in an attempt to provide more “acceptable” rather than honest answers. Furthermore, the data presented here is related to participants’ attributions of the influences on their behaviour. While no differences in opinions or behaviour were identified among the different levels of prescribing behaviour, high prescribers claimed to be medium prescribers, and medium prescribers claimed to be low prescribers during the interview, which could be evidence of social desirability effects. However, the interviewer was blinded to participants’ prescribing practice in order to minimise interviewer biases. Although participants were encouraged to set up the telephone interview for a time and place that was convenient for them, some conducted it from their practice reception, staff room or in-between treating patients. This may have led to the participants feeling less able to disclose certain information.

This study has a number of strengths. Participants’ diversity was evident for a range of variables (Health Board, practice type and size, practice location and deprivation). Furthermore, the use of an iterative process of data collection and analysis allowed us to identify the point at which no new ideas emerged within the TDF domains and thus ensured that data saturation was achieved. A further strength is the study’s theoretical basis, the TDF, which identified barriers and facilitators which could be targeted to improve evidence-based practice. Using such a comprehensive framework ensured that a wide range of influences on the target behaviour was considered, rather than a restricted set of influences that may be explored when research is limited to single theories of behaviour. The TDF also has linked the interview results to intervention functions from the Behaviour Change Wheel framework for designing future interventions. The principles applied here are generalizable to a range of other health services research.

## Conclusions

Antibiotics are widely used by GDPs for managing bacterial infections and often prescribed without conjunctive local measures even although most infections can be treated successfully by local measures. The results from this interview study are important (i) to help understand the barriers to the implementation of evidence-based practice for managing bacterial infections among GDPs and (ii) as a basis for intervention development. A pragmatic approach will be needed to address the modifiable barriers appropriately in future intervention studies in order to change dentists’ inappropriate prescribing behaviour.

## Additional file

Additional file 1:
**Coding guide.** The coding guide used to define TDF domains.

## Abbreviations

A&F: audit and feedback
BCW: Behaviour Change Wheel
CPD: continuing professional development
GDPs: general dental practitioners
ISRCTN: International Standard Randomised Controlled Trial Number
NHS: National Health Services
R&D: Research and Development
RAPiD: Reducing Antibiotic Prescribing in Dentistry
RCT: randomised controlled trial
SDCEP: Scottish Dental Clinical Effectiveness Programme
SIMD: Scottish Index of Multiple Deprivation
TDF: Theoretical Domains Framework
TRiaDS: Translation Research in a Dental Setting
UK: United Kingdom
