# Supplementary material for: Barriers and facilitators of evidence-based management of patients with bacterial infections among general dental practitioners: a theory-informed interview study
Source: Implement Sci. 2016 Jan 29;11:11. doi: 10.1186/s13012-016-0372-z (PMC4731984; doi:10.1186/s13012-016-0372-z)
Supplement: Additional file 1: — Coding guide. The coding guide used to define TDF domains. [file 13012_2016_372_MOESM1_ESM.doc]

**Additional file 1: Coding Guide**

Coding employed directed content analysis [32] using the Theoretical Domains Framework (TDF) [23].

1) Objective of coding is to identify;

1. What we conclude about each TDF domain (is it a barrier or facilitator to managing patients with bacterial infections using local measures rather than prescribing antibiotics?)

2) Where multiple domains are raised by interviewees within the one utterance, judge what is the main message of the utterance and code based on this

3) When uncertain of domain, go with first hunch and asterisk quote in table to show uncertainty and highlight for team discussion

4) If after discussion, uncertainties remain then utterance to be ‘double-badged’ within more than one domain.

| **TDF Domains** | **Coding Guide** |
| --- | --- |
| **Behavioural regulation** | a)Ways of doing things that relate to pursuing and achieving desired goals, standards or targets (e.g. CPD courses/appropriate payment structure/emergency slots could influence the management of patients with bacterial infections using local measures (LMs) rather than prescribing antibiotics  b)Translating intention into action (e.g. at the individual level – action planning; at the organisational level – upgraded/more specific guidelines related to managing patients with bacterial infections will facilitate conducting LMs rather than prescribing antibiotics) |
| **Social influences** | External pressure from other people/ views of other professions, patients that influence the dentist’s behaviour about how to manage bacterial infections rather than prescribing antibiotics. |
| **Reinforcement** | Whether any financial/non-financial incentives/positive or negative consequents influence dentists behaviour on managing bacterial infections using LMs |
| **Environmental context and resources** | Factors related to the dental setting/environment/person-environmental interactions that influence dentists behaviour about managing bacterial infections- using LMs/prescribing antibiotics (e.g. lack of time) |
| **Beliefs about consequences** | Perceptions about outcomes, and advantages and disadvantages of using LMs rather than prescribing antibiotics (e.g. LMs solve problems as opposed to antibiotics) |
| **Beliefs about capabilities** | a) Perceptions about her/his own competence/self-confidence in performing LMs instead of prescribing antibiotics (e.g. very confident of conducting LMs)  b) Perceptions about control over his/her own behaviour, i.e. whether performing LMs instead of prescribing antibiotics is within or out with his/her control (e.g. difficulties with getting patients numb/time are not enough for conducting LMs)  c) Self-efficacy – a person’s confidence that s/he can employ the skills that are necessary to resist temptation, cope with stress, and mobilize her/his own resources required to meet the demands of a situation. It is the person’s own internal attribution of their ability to do this (e.g. difficult to arrange emergency slots) |
| **Memory, attention & decision processes** | The processes involved, and the factors (e.g. a patient’s circumstances) taken into account, when they make the decision to treat bacterial infections using LMs and/or antibiotics |
| **Optimism** | The confidence expressed that things will happen for the best i.e. that LMs will successfully manage infection. Pessimism also coded within this domain i.e. managing bacterial infection using LMs will end in disaster during holidays/weekends, hence antibiotics were prescribed |
| **Emotion** | Feelings or affect about conducting LMs instead of prescribing antibiotics (e.g. Felt anxious about patient’s situation and therefore prescribed antibiotics; LM is stressful) |
| **Knowledge** | Existing procedural knowledge, knowledge about guidelines/SDCEP, knowledge about evidence/how to treat bacterial infections using LMs, and antibiotics |
| **Skills** | Competence, ability required/proficiency acquired through practice to manage patients with bacterial infections using LMs than prescribing antibiotics |
| **Goals** | Priorities, importance, commitment to a certain course of actions or behaviours, intentions |
| **Intentions** | A conscious decision to perform a behaviour, stability of intentions (e.g. always intends to perform LMs first but if the problem is not solved, only then antibiotics are prescribed) |
| **Social/Professional role & identity** | Dentists’ expressions about their own professional identity/job/role/professional boundaries and comparisons about their role with that of other professionals (e.g. treating with LM instead of antibiotics is part of my job as an NHS dentist) |
